# Newly Diagnosed HIV in an Octogenarian Woman: A Case Report on Delayed Diagnosis in Vulnerable Populations

**DOI:** 10.1002/ccr3.70654

**Published:** 2025-07-28

**Authors:** HeeKyoung Choi, Myung Hee Chang, Heun Choi, Wooyong Jeong

**Affiliations:** ^1^ Department of Infectious Diseases National Health Insurance Service Ilsan Hospital Ilsan South Korea; ^2^ Department of Oncology‐Hematology National Health Insurance Service Ilsan Hospital Goyang South Korea

**Keywords:** 80 and over, aged, health literacy, HIV infections, lymphoma

## Abstract

HIV infection in elderly individuals is often under recognized due to stigma, low clinical suspicion, and communication barriers. We present the case of an 85‐year‐old woman from a rural area who was diagnosed with HIV during staging workup for newly diagnosed diffuse large B‐cell lymphoma. Her HIV viral load was 70,650 copies/mL and CD4 count was 83 cells/mm^3^ at diagnosis. She was illiterate, socially isolated, and had no known risk factors. Antiretroviral therapy was initiated with Biktarvy, resulting in gradual immunologic improvement and viral suppression. This case highlights the need to consider HIV testing in older adults with social vulnerability, even when classical risk factors are absent.

**Summary**

- The diagnosis of human immunodeficiency virus (HIV) infection in elderly patients is uncommon and poses unique challenges.
- This case demonstrates delayed HIV diagnosis in an 85‐year‐old woman with lymphoma and illiteracy, highlighting the need for tailored patient communication strategies in vulnerable populations.

**Abbreviations**
ART: antiretroviral therapy
HIV: human immunodeficiency virus

## 1 Introduction

The diagnosis of Human immunodeficiency virus (HIV) infection in elderly patients poses unique challenges [1, 2, 3] In the United States, the highest relative increase in new HIV diagnoses from 2015 to 2019 occurred in those aged 50 and older, with nearly half presenting with advanced immunosuppression [2]. Compared to younger individuals, those over 50 are estimated to be two to four times more likely to experience delayed HIV diagnosis [2, 4]. Newly diagnosed cases in octogenarians remain rare and only a few case reports have been described [5, 6, 7, 8]. This case underscores the importance of considering HIV in differential diagnoses for elderly patients with immunosuppression and malignancies, particularly in those with social isolation and low health literacy. By describing a rare case of HIV diagnosis in an octogenarian without identified risk exposures, this report adds to the limited literature on delayed HIV recognition in older adults and highlights gaps in current screening practices.

## 2 Case History/Examination

An 85‐year‐old woman was referred to the infectious disease department from the hematology department at the National Health Insurance Service Ilsan Hospital, a public secondary‐care general hospital, following a positive HIV confirmatory test. She was asymptomatic at the time of referral. The patient appeared chronically ill but had stable vital signs and no specific abnormalities on physical examination.

Her history at our hospital began in March 2018 when she first presented to the gastroenterology department with right upper quadrant discomfort. The patient had no prior history of chronic illness or surgery. She had not received care at other medical institutions and was not taking any regular medications. Initial evaluations showed elevated liver enzyme levels, but tests for antinuclear antibody, antimitochondrial antibody, hepatitis B, and hepatitis C were negative. She was diagnosed with hepatitis and was regularly followed every 3–4 months with blood tests or abdominal sonography. A gastric ulcer was detected in a national health check‐up program in late 2023. She mentioned this finding during her regular outpatient visit in 2024, which prompted repeat esophagogastroduodenoscopy and biopsy at our hospital in April 2024. The biopsy results revealed diffuse large B‐cell lymphoma. She was referred to the hematology department in May 2024, where a baseline study, including HIV testing, was performed before starting first‐line chemotherapy. The HIV test was conducted as part of routine baseline screening before initiating chemotherapy, not due to clinical suspicion. The HIV test returned positive, and she was referred to the infectious disease department. Upon referral, further investigations were conducted. The CD4 lymphocyte count was 83 cells/mm^3^, and the HIV viral load was 70,650 copies/mL.

## 3 Differential Diagnosis, Investigations, and Treatment

Her husband, who passed away over 20 years ago due to ischemic heart disease at a university hospital, was her only known sexual partner. He had no other significant illnesses before his death. While it is unclear if he was tested for HIV, the family mentioned that he underwent various procedures and tests during his hospital stay with ischemic heart disease, making it unlikely that he had undiagnosed HIV. Both of her sons tested negative for HIV. The patient and her family confirmed that she had never received any blood transfusion before her lymphoma diagnosis. The patient's transmission route remains unclear. The patient had been living alone in a rural area since her husband's death over two decades ago, with limited contact with her two adult sons, who reside separately. She reported no history of sexual activity after her husband's passing and denied any past blood transfusions, injection drug use, acupuncture, or tattooing. She had not undergone any surgical procedures or hospitalizations prior to her lymphoma diagnosis. While she underwent routine upper endoscopy in 2023 as part of a national health check‐up, there were no reports of infection control breaches at the facility. Given her significantly reduced CD4 count (83 cells/mm^3^) and high HIV viral load (70,650 copies/mL) at the time of diagnosis, the infection is presumed to have been acquired several years earlier. During the interview in the clinic, the patient indicated that she was unfamiliar with HIV. Also, the process of obtaining informed consent revealed that she was illiterate.

Due to her longstanding illiteracy and social isolation, the accuracy and completeness of self‐reported exposure history may have been limited. Although the precise transmission route could not be confirmed, sexual transmission remains a plausible explanation.

The patient did not present with overt memory disturbances, confusion, or behavioral changes suggestive of cognitive impairment at the time of evaluation. There were no clinical indications of dementia, and she was able to engage in meaningful conversation with the medical team despite her inability to read or write. Therefore, her limited health literacy and long‐standing social isolation, rather than cognitive decline, were considered the primary barriers to accurate and complete history taking.

She was started on Biktarvy (a combination of bictegravir, emtricitabine, and tenofovir alafenamide) as an antiretroviral therapy (ART). Biktarvy was selected for its tolerability in older adults and low potential for drug–drug interactions [9] The patient received this regimen alongside systemic chemotherapy, and any adverse effects or clinically significant interactions were not observed. In addition, she received trimethoprim/sulfamethoxazole for the prevention of opportunistic infections.

## 4 Outcome and Follow‐Up

She showed good adherence to ART, tolerated chemotherapy well, and remained clinically stable during follow‐up. She showed steady immunologic and virologic improvement. Her CD4 count increased, and viral load was suppressed. Progress is summarized in Table 1.

**TABLE 1:** Trends in CD4 count and viral load after antiretroviral treatment initiation.

Week	CD4 count (cells/mm^3^)	Viral load (copies/mL)
4	273	40
10	273	< 20
17	268	23
21	429	< 20

## 5 Discussion

This case highlights several important considerations for the diagnosis of HIV in elderly patients, particularly those from underserved populations. The patient did not fit the typical high‐risk profile for HIV due to her age and sexual activity, which contributed to the delayed diagnosis.

Newly diagnosed HIV infection in octogenarians has been rarely reported [5, 6, 7, 8]. Reported cases describe delayed diagnosis [5, 6], non‐specific symptoms [6], absence of clearly identified risk factors [7], and HIV detection during the treatment of opportunistic infection or malignancy [6, 7] (Table 2).

**TABLE 2:** Summary of HIV cases newly diagnosed in the octogenarian.

Study	Age/sex	Reported transmission rouge	Initial manifestation	CD4 at diagnosis (cells/mm^3^)	HIV viral load (copies/mL)	Notes
Laszlo et al. [7]	81/F	Unknown	Disseminated tuberculosis	18	> 1,000,000	No clear risk factor, diagnosed during an opportunistic infection
Cloud et al. [5]	83/M	Heterosexual (presumed)	Weight loss, unexplained anemia and leucopenia	106	173,000	No clearly documented exposure
Bąkowska et al. [8]^a^	81/M	Bisexual	Prostatic carcinoma	363	> 100,000	Referenced by Chen et al. (2015); full text not accessible
Chen et al. [6]	83/M	Heterosexual (presumed)	CMV pneumonitis, acute respiratory failure	42	5,902,000	Diagnosed during ICU care
Present case	85/F	Unknown	Asymptomatic at referral; prior lymphoma diagnosis	83	70,650	Found during pre‐chemotherapy evaluation

^a^ This case was cited in Chen et al. (2015) but could not be accessed in full text for verification.

Although sexual contact is the main mode of HIV transmission in older patients [10], age‐related stigma and misconceptions about HIV risk play a significant role in the late diagnosis of the disease in older adults [3]. Many healthcare providers and patients themselves often do not consider HIV as a possible diagnosis for elderly individuals, leading to delays in testing and diagnosis. This case aligns with emerging perspectives in geriatric HIV medicine, which call for incorporating frailty assessment and comprehensive geriatric approaches in managing older HIV‐positive individuals [1].

Health literacy is a crucial factor for HIV‐related health information, treatment adherence, and health outcomes [11]. In this case, the patient's inability to read likely hindered her understanding of health risks and HIV. It prevented her from seeking timely medical care. Illiteracy further complicates matters, limiting patients' ability to access and comprehend health information.

Although the patient had medical contact over several years, HIV testing was only performed during pre‐chemotherapy evaluation. Multiple factors likely contributed to this diagnostic delay. On the patient level, longstanding illiteracy and social isolation may have limited her ability to recognize symptoms or communicate effectively with healthcare providers. On the provider level, low clinical suspicion of HIV in elderly patients, particularly those without known risk behaviors, likely delayed testing. Implicit biases and stereotypes may have further compounded under‐screening. Healthcare providers may subconsciously assume that older adults are not sexually active or are unlikely to engage in behaviors associated with HIV transmission. Such perceptions can lead to omission of risk assessment, avoidance of sensitive topics during consultation, and failure to offer HIV testing. In this case, testing was conducted only as part of a routine pre‐chemotherapy evaluation, not due to proactive clinical consideration. At the system level, HIV screening is typically focused on individuals aged 13–64. There are no national guidelines encouraging routine testing in older adults, even those with vulnerability indicators. These overlapping barriers demonstrate the need for broader screening considerations in aging populations. This case exemplifies how diagnostic delays in older adults can result from a convergence of patient‐related, provider‐related, and systemic barriers, each reinforcing the other. Addressing this diagnostic inertia requires both provider education and guideline adaptation to account for vulnerability.

Even in countries like South Korea, where healthcare access is generally good, vulnerable populations can fall through the cracks, emphasizing the need for proactive and comprehensive screening measures to ensure early diagnosis and treatment.

Routine HIV screening is generally recommended for individuals aged 13 to 64 [12]. This can lead to missed early diagnosis and intervention opportunities in the elderly population. In this case, the patient's HIV infection was only discovered incidentally during an investigation for lymphoma, highlighting the need for broader screening guidelines that include older adults, particularly those in socioeconomically disadvantaged situations.

According to national HIV reports in South Korea, approximately 1.8% of new HIV cases in 2022 were individuals aged 70 and above [13]. However, these reports aggregate data for all individuals aged 70 and above, making it difficult to ascertain the specific incidence rate among octogenarians and older patients. This lack of specific data underscores the rarity and potential under‐recognition of HIV in this very elderly subgroup.

Moreover, this case demonstrates that elderly patients can achieve significant immunologic and virologic responses to ART despite their age. These findings emphasize the importance of the timely initiation of ART in elderly populations, as age should not preclude the potential for effective treatment outcomes. While this short‐term response is encouraging, longer follow‐up is necessary to assess the treatment's success and sustainability fully.

Ethical considerations are particularly important when managing HIV in vulnerable elderly patients. In this case, the patient had no prior knowledge of HIV and limited health literacy, which required tailored communication to ensure informed understanding and consent. Her verbal consent to treatment and publication was obtained through a simplified explanation process involving both medical staff and family.

Additionally, the diagnosis carried a high risk of stigma, especially in the context of South Korea's conservative cultural norms regarding sexuality and aging. Maintaining confidentiality was an essential component of care. These ethical challenges highlight the need for more structured protocols to support HIV screening and disclosure in elderly populations, particularly those with reduced autonomy or limited access to health information.

Beyond clinical management, the psychosocial impact of an HIV diagnosis in elderly patients should not be underestimated. This patient had been living alone in a rural area, with limited social support and low health literacy. Although the patient did not exhibit overt emotional distress, the diagnosis may still have carried an underlying psychological burden, particularly in the context of age‐related stigma. In cultures where sexuality in older age is rarely acknowledged, HIV infection can be associated with self‐stigmatization. These factors may affect mental well‐being and quality of life, even in the absence of diagnosable psychiatric conditions.

This case report has several strengths. First, it contributes to the limited but growing literature on HIV diagnosis in octogenarians, a population often excluded from HIV screening guidelines. Second, it highlights the intersection of clinical, social, and structural vulnerabilities in an elderly patient with limited health literacy, rural isolation, and no known risk behaviors, offering important insights for clinicians managing similar patients.

However, this report also has limitations. As a single case, its findings are not generalizable to all elderly individuals with HIV. Additionally, the patient did not disclose specific behavioral risk factors, which limits the ability to definitively understand the transmission route. Lastly, longer‐term clinical outcomes (beyond virologic and immunologic responses) were not captured due to the limited follow‐up period.

Overall, this case underscores the necessity for increased awareness of HIV among elderly populations, particularly those with poor health literacy backgrounds. While universal HIV screening beyond age 64 may not be necessary, this case illustrates the importance of considering screening in older adults with overlapping social and contextual vulnerabilities. Elderly individuals with limited health literacy, social isolation, or cultural constraints that may hinder open disclosure of past exposures should prompt heightened clinical awareness. When such patients access care, it may be appropriate to consider HIV screening, especially in the context of immunologic abnormalities or delayed diagnoses.

## Author Contributions

**HeeKyoung Choi:** conceptualization, writing – original draft. **Myung Hee Chang:** writing – review and editing. **Heun Choi:** writing – review and editing. **Wooyong Jeong:** writing – review and editing.

## Consent

Written informed consent for publication was not obtained due to the patient's illiteracy, as she could not provide a written signature. However, the Institutional Review Board of NHIMC (IRB No. NHIMC IRB 2024‐12‐019) reviewed this case and approved a waiver of written consent specifically for publication. Following the IRB‐approved procedure, a thorough verbal explanation was provided to the patient regarding the nature and purpose of the publication, and verbal informed consent for publication was obtained and recorded in the patient's medical chart.

## Conflicts of Interest

The authors declare no conflicts of interest.

## Data Availability

The data that support the findings of this study are available on request from the corresponding author. The data are not publicly available due to privacy or ethical restrictions.
